# Unexpected Activity: Evidence for Obesogenicity of a BPA Metabolite

**DOI:** 10.1289/ehp.123-A303

**Published:** 2015-12-01

**Authors:** Wendee Nicole

**Affiliations:** Wendee Nicole has written for *Discover*, *Scientific American*, and other publications.

Bisphenol A (BPA) has been implicated as an obesogen, a compound that alters lipid metabolism, promoting development of adipocytes (fat cells) and accumulation of fat.^1^^,^^2^ BPA is quickly converted in the body to its main metabolite, BPA β-D-glucuronide (BPA-G), which has been thought to be biologically inactive.^3^ But a study reported in this issue of *EHP* indicates that BPA-G, like its parent compound BPA, can induce precursor cells called preadipocytes to develop into mature fat cells.^4^

In one experiment the researchers treated mouse preadipocytes with 10 μM of BPA-G. This resulted in a significant increase in fat accumulation and stimulated protein expression of three adipogenic markers—lipoprotein lipase, aP2, and adipsin—in the mouse cells. In experiments with human preadipocytes, they found that both 0.05 and 0.25 μM BPA-G stimulated fat cell differentiation as determined by aP2 protein levels. However, co-exposure with fulvestrant—an estrogen-receptor (ER) antagonist used to treat some breast cancers—inhibited the changes that were seen with BPA-G alone.^4^

**Figure:**
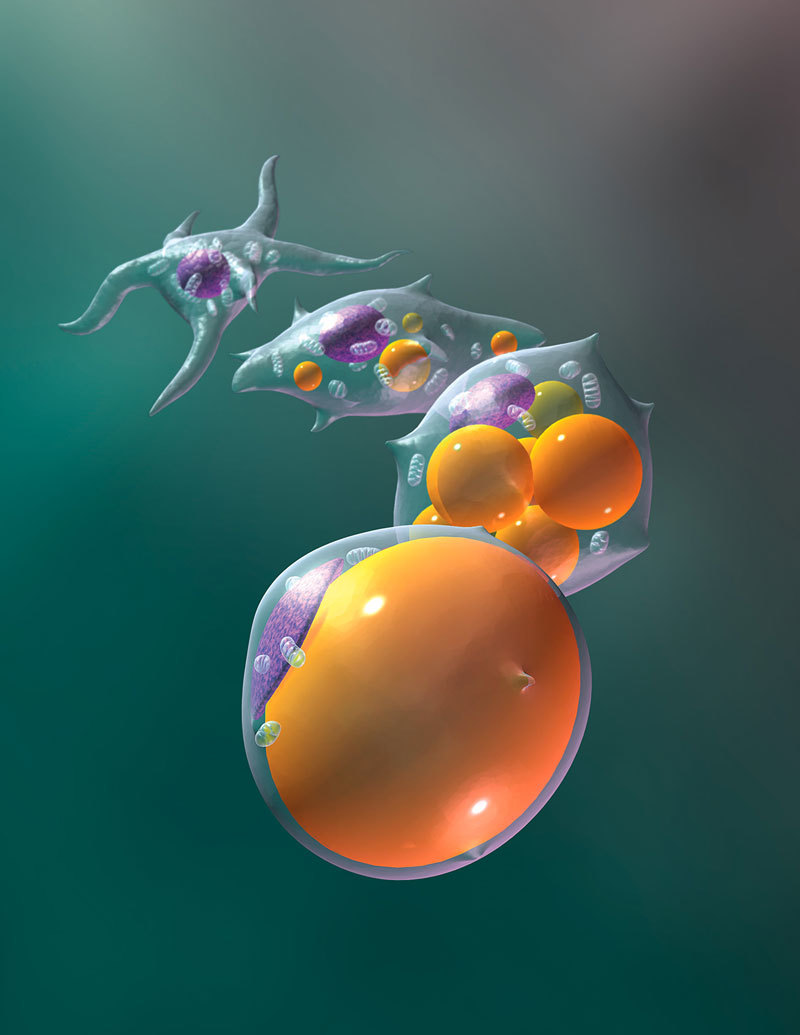
Preadipocytes are a renewable source of new adipocytes (fat cells), which accumulate and store lipids for use as energy. Obesogens spur more preadipocytes to differentiate into fat cells and cause mature fat cells to accumulate more lipids. © Gary Carlson/Science Source

The authors also observed that BPA-G did not have direct estrogenic activity, unlike its parent compound BPA. At the same time, its effects on adipogenesis were inhibited by an ER antagonist. The authors suggest this could indicate an effect through nonclassical ER signaling—in other words, an effect that does not involve a direct interaction between the ER and DNA—but they acknowledge that other unidentified mechanisms also are possible.^4^

The authors included human cells in their experiments to assess whether there were differences in how different species respond. “As can be seen in the paper, the human cells were also responsive,” says team leader Ella Atlas, a research scientist with Health Canada’s Environmental Health Science and Research Bureau. “We were surprised to find that BPA-G was active and that it seemed even more potent in the human preadipocytes.”

BPA is used in a wide variety of products, including polycarbonate plastics, food and beverage containers, and coated papers (e.g., thermal receipts).^5^ The U.S. Food and Drug Administration (FDA) currently considers BPA safe at the levels occurring in food and food packaging.^6^ FDA researchers have reviewed the new study, says spokeswoman Marianna Naum, and have determined that the results do not provide a basis for altering the FDA’s assessment at this point. “This assessment remains consistent with Health Canada’s own assessment of BPA safety^7^ as well as that of the European Food Safety Authority^8^,” Naum says.

But the findings raise important questions that must be addressed, given the ubiquity of exposure to BPA.^5^ “This is an interesting paper that demonstrates a possible *in vivo* function for BPA-glucuronide which has heretofore been viewed as an inactive metabolite,” says Bruce Blumberg, a biology professor at the University of California, Irvine, who was not involved with the study. “If these authors are correct, then the argument that BPA is immediately rendered inactive by conjugation to BPA-glucuronide after ingestion, making it harmless, is inaccurate and misleading.”
